# Determinants of nutritional status among pregnant women in East Shoa zone, Central Ethiopia

**DOI:** 10.3389/fnut.2022.958591

**Published:** 2022-12-15

**Authors:** Ermias Bekele Wakwoya, Tefera Belachew, Tsinuel Girma

**Affiliations:** ^1^Department of Nutrition and Dietetics, Jimma University, Jimma, Ethiopia; ^2^Department of Pediatrics and Child Health, Jimma University, Jimma, Ethiopia

**Keywords:** nutrition, determinants, pregnant women, undernutrition, Ethiopia

## Abstract

**Background:**

Undernutrition is an insufficient intake of energy and nutrients to meet an individual's needs to maintain good health. Undernutrition during pregnancy severely affects the health of mothers and her baby. Globally it contributes directly or indirectly to 3.5 million maternal deaths annually. This study aimed to determine the level of undernutrition and identify factors associated with undernutrition among pregnant women attending public health facilities in the East Shoa Zone, Central Ethiopia.

**Methods:**

Institution-based cross-sectional study was conducted among 472 randomly selected pregnant women from June to August 2021. Sociodemographic, obstetrics, and knowledge related data were collected using a structured interviewer-administered questionnaire, and maternal nutritional status was measured using the Mid Upper Arm Circumference (MUAC). The collected data were entered to EPI-info version 3.5.4 and then exported to SPSS for windows version 26.0 software for analysis. Multivariable regression analysis was fitted to identify determinants of undernutrition. An adjusted odds ratio with 95% confidence intervals and a *p*-value < 0.05 was considered a statistically significant.

**Results:**

The prevalence of undernutrition among pregnant women was 13.9% [95% CI: 11.0–17.4]. On multivariable logistic regression model after adjusting background variables, wealth (AOR: 4.9, 95% CI 1.34–18.20), women's decision making power (AOR: 3.31, 95% CI 1.18–7.79), and nutritional counseling (AOR: 3.53, 95% CI 1.29–9.60) were independently associated with nutritional status of pregnant women.

**Conclusion:**

Findings indicated that significant number of pregnant women in the study were undernourished. Higher wealth index, nutritional counseling, and women's decision-making power were inversely associated with undernutrition. The findings imply the need for economic empowerment of women, enhancing decision-making ability of women and routine and consistent nutritional counseling to decrease undernutrition among pregnant women.

## Introduction

The first 1,000 days (the period from conception through the first 2 years after birth) is a critical period for the future growth and development of a child (1). Pregnant women should get optimum nutrients for good perinatal outcomes. Taking the right balance of micronutrients at an early stage of pregnancy prevents complications due to malnutrition (2, 3). Undernutrition, energy and nutrients intake insufficient to maintain good health (4), contributes to 3.5 million global maternal deaths annually (5). It remains a significant public health problem in Africa, especially in sub-Sahara countries (6).

Undernutrition during pregnancy severely affects physical and mental health of children. There is a 64 percent probability that women delivering a low birth weight (LBW) infant would have a lower MUAC level than those delivering a normal birthweight infant (7). A systematic review conducted by Han et al. revealed that women with undernutrition had more than 60% increased risk of giving birth to LBW babies (8).

Pregnant women's knowledge about nutrition during pregnancy is a critical nexus in good pregnancy outcomes and improving children's nutritional status (9). Nutritional knowledge and attitude are essential for good dietary practices and are, thus, potential targets for appropriate planning of nutrition interventions for vulnerable population groups like pregnant and lactating women (10–12).

In Ethiopia, undernutrition among pregnant women ranges from 21.8 to 43.1%, with a higher prevalence among rural women (13, 14). Factors that are associated with maternal undernutrition include young age, low educational attainment, low socio-economic status, large family size, low income, poor women's decision-making power, household food insecurity, low nutrition knowledge, residence, dietary diversity, absence of latrine, and unintended pregnancy (13–16).

Risk factors for undernutrition vary by context due to differences in socioeconomic characteristics, culture, ethnicity, and geographical location. The magnitude of undernutrition and associated factors among pregnant women in East Shoa zone are not known. Therefore, this study aimed to determine the level of undernutrition and identify factors associated with undernutrition among pregnant women attending public health facilities in the East Shoa zone, Oromia Region, Ethiopia.

## Methods and materials

### Study area and period

According to the East Shoa zone health office, the health service delivery is organized under 3 hospitals, 59 health centers, and 290 health posts. All the facilities are expected to provide maternal and neonatal health care services based on the National Essential Health Services Packages (EHSP) for different levels of health care. According to the zonal report of 2020, the facilities in the zone provided antenatal care service (ANC) for more than 54,408 pregnant women. The food production system in the district is characterized by mixed crop-livestock farming, with predominant crop production. The community is also known for cultivating different fruits and vegetables, which are considered cash crops. Teff is the principal crop produced in the area. This study was conducted from June to August 2021.

### Study design and population

Institution-based cross-sectional study design was used. All pregnant women who came for ANC service at selected health institutions in Eastern Shoa Zone during the study period were considered the study population.

### Inclusion and exclusion criteria

Pregnant women living in Eastern Shoa Zone and attending ANC service at the selected health center during the study period were given a chance to be included in the study. Severely sick women were not eligible.

### Sample size and sampling procedure

The sample size was calculated by using OpenEpi version 3.01 software (OpenSource.org/licenses). The minimum sample size was calculated using a single population proportion formula. Assuming standard error corresponding to a 95% confidence level (Z) = 1.96, the proportion of undernourished pregnant women from the previous study (*p* = 19.1), (17), margin of error (d) = 5% and design effect 2, the estimated sample size was 475. Multistage stratified sampling techniques was used to select study participants. Of a total of health centers in the east Shoa zone, six health centers from urban and eight health centers were included from rural kebele by using the lottery method. The total sample size was allocated to each health center based on the number of women who visited the health center in the preceding year in the same month. Finally, a systematic random sampling technique was used to select participants by following the K^th^ value. The K^th^ value was calculated by taking the total number of pregnant women on ANC during the study period and dividing it by the sample size, and it was found to be three. Then, lottery methods was used to choose the first case within the interval, which turned out to be 1.The first comer was considered as the first participants, and participants who came at the third interval were interviewed until the determined sample size was achieved.

### Measurement and data collection procedure

A structured and pretested questionnaire was used to collect information on sociodemographic and obstetrics characteristics, women autonomy, Household Food Insecurity Access Scale (HHFIAS), and knowledge of women. The data collectors were trained nurses and midwives recruited for this study. Participants were invited to the survey when they came to receive ANC service at the selected health institution. All the participants completed the survey questions.

Mid upper arm circumference of pregnant women was measured by using inelastic MUAC tape. The midpoint of the left upper arm was located by flexing the women's elbows to 90^0^ with the palm facing upwards and the midpoint between acromion to olecranon processes was marked. After this, measuring tape was placed around the arm at the midpoint. Two measurements was taken and reported to the nearest 0.1 cm. Women with MUAC < 22 cm were considered undernourished, and ≥ 22 cm were considered well-nourished (7). The household food security status in the past 4 weeks before data collection was assessed by using the Food Insecurity Access Scale (HFIAS) measurement tool. The score was calculated for each household by summing up the nine food insecurity-related conditions' frequency of occurrence. A household that obtained < 2 scores were considered food secured and those that obtained ≥ 2 scores were considered food insecure (18). Women's decision-making power was assessed using six questions adapted from previous literature (19). For each question, three options were presented, and one score was given when a decision was made by the woman alone or jointly with her husband, or zero was given if the decision did not involve women. Gravidity is number of times that a women get pregnant. Parity is the number of times that a woman had given birth to a fetus with a gestational age of 24 weeks or more, regardless of the child was born alive or was stillbirth.

A total of 16 questions focusing on nutrition knowledge were presented to participants of this study. For knowledge questions, respondents with an average score greater than or equal to mean score were categorized as having adequate knowledge about nutrition during pregnancy and respondents with average score less than mean value were classified as having inadequate knowledge (20).

### Data quality assurance

To ensure the quality of the data, local languages were used for understanding of the questions. In addition, pre-test of research instruments and thorough training of data collectors and supervisors were done before the actual data collection. The supervisors provided on-site support to data collectors daily. All completed questionnaires were collected by respective supervisors and checked overnight to ensure completeness and consistencies. Regular meetings were held to provide feedback on issues of concern identified from data collected the next day.

### Data processing and analysis

The data were entered to EPI-info version 3.5.4 and then exported to SPSS version 26.0 software for analysis. Binary logistic regression was used to check the association between explanotory variables and undernutrition. All variables with a *p*-value < 0.25 in bivariate analyses remained in the model as potential confounders for multivariable analysis. Hosmer and Lemeshow's goodness-of-fit test was performed to assess whether the required assumption was fulfilled, and variance inflation factors were checked to assess for multi-collinearity. The strength of association was expressed as adjusted odds ratio with 95% confidence intervals. A *p*-value < 0.05 was considered a statistically significant association with undernutrition. Multicolliniarity was checked using standard error of >2.

## Results

### Characteristics of respondents

Of 475 participants approached, 472 (99.4%) responded. The mean age of the respondents was 25.8 (SD ± 4.6) years, 465(98.5%) were married, 235(49.8%) Orthodox Christians, and 296 (62.7%) from Oromo ethnic group (Table 1). One-fourth, 119 (25.2%) of the respondents were unable to read and write, followed by those who attended primary education 117(24.8%) (Table 1).

**Table 1:** Socio-demographic characteristics of pregnant women attending ANC service in East Shoa zone, Ethiopia, from June to August 2021.

**Variables (*N* = 472)**	**Frequency**	**Percent**
**Age, years**		
16–24	214	45.3
25–34	238	50.4
≥ 35	20	4.2
**Marital status**		
Married	465	98.5
Single	6	1.3
Divorced	1	0.2
**Religion**		
Orthodox Christian	235	49.8
Muslim	152	32.2
Protestant	76	16.1
Catholic	9	1.9
**Ethnicity**		
Oromo	296	62.7
Amhara	117	24.8
Tigray	11	2.3
Gurage	35	7.4
Others	13	2.8
**Educational status**		
Unable to read and write	119	25.2
Able to read and write	103	21.8
1–8 grade	117	24.8
9–12 grade	72	15.3
College and above	61	12.9
**Occupation**		
Housewife	321	68
Government employee	46	9.7
Private employee	67	14.2
Daily laborer	16	3.4
Merchant	22	4.7
**Husband's educational status**		
Unable to read and write	56	11.9
Able to read and write	91	19.3
1–8 grade	127	26.9
9–12 grade	104	22
College and above	94	19.9
**Husband's occupation**		
Government employee	80	16.9
Private employee	190	40.3
Daily laborer	93	19.7
Merchant	65	13.8
Unemployed	44	9.4
**Wealth**		
Poorest	46	9.7
Poor	55	11.7
Middle	247	52.3
Rich	73	15.5
Richest	51	10.8

More than two-thirds of respondents' maximum gravidity was two, and 80% gave birth once or twice. For the current pregnancy, 224(47.5) were in the third trimester, 431(91.3%) was planned and 207(84.8%) have nutritional counseling during their ANC follow-up. Of the respondents, 246 (52.1) took 21–30 iron-folate pills in the preceding 1 month, and the primary reason for missing the pills was forgetfulness 144 (30.5%) (Table 2).

**Table 2:** Obstetrics and other related characteristics of pregnant mothers attending ANC service in East Shoa zone, Ethiopia, from June to August 2021.

**Variables (*N* = 472)**	**Frequency**	**Percent**
**Gravidity**		
1–2	319	67.6
3–4	134	28.4
≥ 5	19	4.0
**Parity (*****N*** **=** **287)**		
1–2	229	79.8
3–4	45	15.7
≥ 5	13	4.5
**Gestational age**		
≤ 14 weeks	41	8.9
15–27 weeks	206	43.6
≥ 28 weeks	224	47.5
**Current pregnancy planned**		
Yes	431	91.3
No	41	8.7
**Number of current ANC visit**		
First	177	37.5
Second	168	35.6
Third	77	16.3
Fourth	50	10.6
**Received nutritional counseling currently (*****N*** **=** **244)**		
No	37	15.2
Yes	207	84.8
**Iron-folate pill taken last month (*****N*** **=** **314)**		
≤ 10	158	33.5
11–20	68	14.4
21–30	246	52.1
**Reason for not taking iron-folate (*****N*** **=** **342)**		
Side effects	23	6.7
Forgetfulness	144	42.1
Pills are too many	11	3.2
Test is bad	11	3.2
Didn't missed the pills	153	44.7

### Household food security

**Figure 1:**
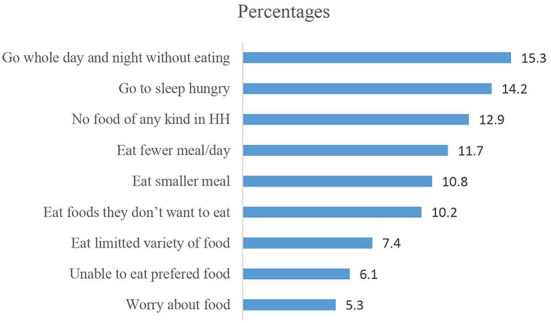
Distribution of household food insecurity based on the HIFAS scale among pregnant women attending ANC in East Shao zone, Ethiopia, from June to August 2021.

The majority of the participants, 374 (79.2%), were from food secured households. Seventy-two (15.3%) and 67(14.2%) of women were either unable to prefer food or ate a limited variety of food at least once in 4 weeks preceding the study period. A significant proportion of women, 61(12.9%), ate fewer meals per day, which happened at least once a month before the data collection period (Figure 1).

### Knowledge of mothers on source of important nutrients

The knowledge assessment revealed that 150 (32.8%) women had poor knowledge about appropriate nutrition during pregnancy; 263 (55.7%), mentioned meat, poultry, and fish as a component of a balanced diet. The participants cited preventing pregnancy complications, 153(32.4), and brain and cognitive development, 179 (37.9%), as the significant advantages of a balanced diet. On the other hand, anemia 178 (37.7%) and LBW 117(24.8%) were the negative consequences mentioned by the respondents. Almost half of the study participants, 232(49.2%), know that they have to use iodized salt while 120 (25.4%) have no idea about the importance of iodized salt and 143 (30.3%) didn't know why iron folate pills are given (Table 3).

**Table 3:** Knowledge of pregnant mothers attending ANC service on optimal nutrition during pregnancy in East Shoa zone, Ethiopia, from June to August 2021.

**Variables *N* = 472**	**Frequency**	**Percent**
**Components of balanced diet**		
Meat poultry and fish	263	55.7
Eggs	45	9.5
Fruits	91	19.3
DGLV	112	23.7
Diary	101	21.4
Pulses	90	19.1
Grains	105	22.2
Roots and tubers	51	10.8
I don't know	90	19.1
**Benefits of balanced diet for woman**		
Prevent pregnancy complications	153	32.4
Prevent delivery complications	50	10.6
Prevent anemia	75	15.9
Prevent from disease	152	32.2
Energy storage for lactation	37	7.8
Don't know	104	22.0
**Benefits of balanced diet for fetus**		
For brain and cognitive development	179	37.9
Prevent IUGR	62	13.1
Prevent LBW	106	22.5
Prevent still birth	58	12.3
Don't know	128	27.1
**Consequences of under nutrition for woman**		
Cause anemia	178	37.7
Make her susceptible to disease	141	29.9
Cause delivery complications	84	17.8
Don't know	100	21.2
**Consequences of under nutrition for fetus**		
Low birth weight	117	24.8
Still birth	62	13.1
IUGR	87	18.4
Preterm birth	74	15.7
Impaired cognitive development	64	13.6
**Additional meal per day**		
One	51	10.8
Two	104	22.0
Three	168	35.6
Four	149	31.6
**Type of salt**		
Iodized salt	232	49.2
Non iodized salt	120	25.4
Don't know	120	25.4
**When should a salt added to food**		
At the beginning of cooking	36	7.6
At the middle	104	22.0
At the end but still on fire	205	43.4
When serving food	30	6.4
I don't know	97	20.6
**Use of iron folic acid supplement**		
For cognitive development of fetus	54	11.4
Prevent LBW	32	6.8
Prevent birth	90	19.1
Prevent anemia	153	32.4
Don't know	143	30.3
**What should a woman do if she has IFA related heart burn?**		
Buy and take antacid	28	5.9
Stop taking IFA supplement	36	7.6
Take IFA after meal	43	9.1
Consult HCP	237	50.2
Don't know	128	27.1

Regarding iron, 152 (32.2%) did not know the food sources, and 56 (11.9%) reported meat, poultry, and fish as a source of iron. None of the participants were able to answer a single source of vitamin 147(31.1%) and protein 142 (30.1%). Dairy products were mentioned as sources of calcium by 168 (35.6%) women (Table 4).

**Table 4:** Knowledge of mothers on source of important nutrients in East Shoa zone, from June to August 2021.

**Variables**	**Frequency**	**Percent**
**Sources of iron**		
Meat, poultry, fish	56	11.9
Eggs	11	2.3
Fruits	37	7.8
DGLV	32	6.8
Diary	53	11.2
Pulses	27	5.7
Grains	89	18.9
Roots and tubers	15	3.2
I don't know	152	32.2
**Source of vitamins**		
Meat, poultary, fish	69	14.6
Eggs	8	1.7
Fruits	53	11.2
DGLV	106	22.5
Diary	29	6.1
Pulses	20	4.2
Grains	33	7.0
Roots and tubers	7	1.5
I don't know	147	31.1
**Source of protein**		
Meat, poultry, fish	132	28.0
Eggs	18	3.8
Fruits	27	5.7
DGLV	33	7.0
Diary	59	12.5
Pulses	44	9.3
Grains	11	2.3
Roots and tubers	6	1.3
I don't know	142	30.1
**Source of calcium**		
Meat, poultry, fish	27	5.7
Eggs	7	1.5
Fruits	28	5.9
DGLV	33	7.0
Diary	168	35.6
Pulses	18	3.8
Grains	23	4.9
I don't know	168	35.6

### Nutritional status of pregnant women

In the current study, 405 (85.8%) of the women were well-nourished. Wealth, women's decision-making power, and nutritional counseling were independently associated with the nutritional status of pregnant women (Table 5). Poor women had 4.9 times higher odds of undernutrition compared with rich women (AOR: 4.9, 95% CI 1.3–18.2). Women with poor decision-making power had 3.3 higher odds of undernutrition than their counterparts (AOR: 3.3, 95% CI 1.2–7.8). Pregnant women who haven't received nutritional counseling during ANC follow-up had 3.5 higher odds of undernutrition than the women who received the counseling (AOR: 3.5, 95% CI 1.3–9.6) (Table 5).

**Table 5:** Binary and multivariable logistic regression analysis showing the association of undernutrition among pregnant women attending ANC service in East Shoa zone, Ethiopia, from June to August 2021.

**Variables (*N* = 472)**	**Nutritional status**		**COR (95% C.I)**	**AOR (95% C.I)**
	**Normal**	**Undernourished**		
**Occupation**				
Unemployed	267 (84.0%)	51 (16.0%)	1.77 [0.96–3.26]	2.38 [0.86–5.56]
Employed	139 (90.3%)	15 (9.7%)	Ref	Ref
**Wealth**				
Poor	86 (78.9%)	23 (21.1%)	3.58 [1.52–8.39]^*^	4.9 [1.34–18.20]^*^
Medium	213 (85.9%)	35 (14.1%)	2.19 [098–4.90]^*^	2.32 [0.69–7.81]
Rich	107 (93.0%)	8 (7.0%)	Ref	Ref
**Educational status**				
No formal education	191 (83.4%)	38 (16.6%)	Ref	Ref
Have formal education	215 (88.5%)	28 (11.5%)	0.65 [0.39–1.11]	0.98 [0.43–2.23]
**Women decision making**				
Poor	49 (69.0%)	22 (31.0%)	3.64 [2.01–6.59]^**^	3.04 [1.18–7.79]^*^
Good	357 (89.0%)	44 (11.0%)	Ref	Ref
**Food security**				
Food secured	199 (89.6%)	23 (10.4%)	Ref	Ref
Food unsecured	207 (82.8%)	43 (17.2%)	1.79 [1.04–3.09]^*^	0.37 [0.12–1.18]
**Know protein source foods**				
Incorrect	180 (82.2%)	39 (17.8%)	1.81 [1.07–3.08]^*^	1.09 [0.48–2.49]
Correct	226 (89.3%)	27 (10.7%)	Ref	Ref
**Nutritional counseling**				
No	28 (75.7%)	9 (24.3%)	2.45 [1.03–5.81]^*^	3.53 [1.29–9.60]^*^
Yes	183 (88.4%)	24 (11.6%)	Ref	Ref
**Medical illness**				
No	283 (84.5%)	52 (15.5%)	1.61 [0.86–3.02]	1.62 [0.63–4.19]
Yes	123 (89.8%)	14 (10.2%)	Ref	Ref

* = significantly associated at p–value < 0.05,

** strongly significantly associated at p–value < 0.01, COR, Crude Odds Ratio; AOR, Adjusted Odds Ratio.

## Discussion

This study assessed the nutritional status and associated factors among pregnant women attending ANC follow-up in East Shoa Zone, central Ethiopia. The study revealed that factors like wealth, women's decision-making power, and nutritional counseling were significantly associated with the nutritional status of pregnant women.

The effect of food insecurity appears significant during pregnancy. Because it compromises the quantity and quality of food intake, it causes adverse feto-maternal health outcomes and congenital disabilities (21). We found that 20.8% of women came from food-insecure households in the current study, consistent with the study done in northwest Ethiopia, where 20.6% came from food-insecure households (22). However, the result is lower than the report from a rural community in southern Ethiopia, where 38.6% of participants were from food-insecure households (23). The difference might be due to socioeconomic status, population density, and other geographical characteristics.

Pregnant women need to get a variety of food to meet the high need for energy and minerals due to increased maternal metabolic activity and the growing fetal tissue in her womb. In this study, around one in seven women ate a limited variety of foods at least once in the preceding month. This indicates a significant number of women were facing a challenge to get variety of foods during pregnancy in the study area. Which is similar to the report of previous studies in Ethiopia (22, 24).

Improving the women's knowledge is effective to modify the dietary practices of pregnant women (25). In Ethiopia, previous studies revealed that many pregnant women had poor knowledge of appropriate nutrition for pregnant women (24, 26, 27). Similarly, in our study, nearly one-third of the respondents had poor knowledge about appropriate nutrition during pregnancy. Among this, a significant proportion of participants could not cite a single protein, vitamins or iron food source. This finding pointed out the high need for establishing appropriate monitoring and feedback mechanisms on antenatal nutrition education and counseling for health care providers as this could improve the knowledge of women about appropriate nutrition.

Different interventions have been undertaken to curb the high prevalence of maternal undernutrition in the past years by the Ethiopian Federal Ministry of Health and other stakeholders (28, 29). However, high maternal undernutrition is still a challenge in many parts of the country (15, 23). We found that 14 percent of women were undernourished which is less than what was reported in the previous studies (24, 26, 30). The difference could be due to access to food, socioeconomic status, and awareness about nutrition during pregnancy. Effective implementation of strategies and evidence-based interventions such as; micronutrient supplementation, nutritional counseling, deworming prophylaxis, environmental and personal hygiene promotion could alleviate maternal undernutrition during pregnancy.

Access to variety of foods is possible when the women have enough money to purchase these foods and individuals' purchasing capacity determines the type of foods they consume (31–33). Higher odds of undernutrition observed among poor women in the present study are consistent with the previous study of young pregnant women from Ethiopia (34). In developing countries like Ethiopia, women have less access to jobs and unable to support their daily needs and the needs of their families. Hence, it logically follows that investment in women's economic empowerment would have a beneficial effect on the nutritional status of pregnant women.

Women's decision-making autonomy is the base for women's empowerment, and it ensures that women to have a good dietary diversity (19). Subsequently, having dietary diversity will help the women be in good nutritional status. In the present study, the odds of undernutrition was three times higher among women with poor decision-making power. This finding was supported by evidence from eastern Ethiopia, where the risk of malnutrition increased by more than double in pregnant women with low and medium levels of autonomy than in those who had a high level of autonomy (17). Programs working to improve the nutritional status of pregnant women should focus on increasing women's decision-making power.

Antenatal nutrition counseling and education (NEC) is one of the widely utilized strategies to improve women's nutritional status during pregnancy. NEC's goal is to improve maternal dietary quality by increasing the diversity and amount of foods consumed, and desired weight gain through the consumption of a sufficient and balanced diet. According to Girard &Olude, NEC improved gestational weight gain by 0.45 kg and reduced the risk of anemia by 30 percent (24). Lack of nutritional counseling was also significantly associated with low birthweight (35). In the present study, the odds of undernutrition are 3.5 times higher among women who haven't received nutritional counseling during pregnancy than those who received the counseling.

Though the evidence revealed that nutritional counseling during pregnancy improves the nutritional status of women and birth outcomes, the emphasis given to this intervention in developing countries is still low. For instance, majority of pregnant women who participated in the study done in Addis Ababa believe nutrition counseling provided to expectant mothers is not adequate and is neglected by most stake holders (36). Therefore, healthcare providers and hospital administrators should emphasize nutritional counseling and commit themselves to provide nutritional counseling to all women coming for ANC service.

This study included adequate sample size and assessed a range of determinants based on the constructed conceptual framework. The high response rate in this study minimizes the risk of non-response bias. The study only relied on MUAC to identify the nutritional status of women, and the limitations of this measurement could affect the findings of this study.

## Conclusion

Findings indicated that a significant number of pregnant women in the study area were undernourished. It was also observed that higher wealth index, nutritional counseling, and women's decision-making power were inversely associated with undernutrition. Based on these findings, women's economic empowerment and increasing decision-making ability through direct and indirect interventions were recommended to decrease the magnitude of undernutrition. Additionally, routine and consistent nutritional counseling, establishing regular monitoring, and feedback mechanism are recommended to improve the nutritional status of women.

## Data availability statement

The raw data supporting the conclusions of this article will be made available by the authors, without undue reservation.

## Ethics statement

The studies involving human participants were reviewed and approved by Institutional Review Board (IRB) of Jimma University, College of Health Sciences. The patients/participants provided their written informed consent to participate in this study.

## Author contributions

EW, TB, and TG participated in the conception, study design, acquisition of data, analysis, interpretation, drafted, and critically reviewed the article. All authors have agreed on the journal to which the article will be submitted, reviewed, and agreed on all versions of the article before submission and during revision. All authors agree to take responsibility and be accountable for the contents of the article.
